# Resting Full-Cycle Ratio versus Fractional Flow Reserve: A SWEDEHEART-Registry-Based Comparison of Two Physiological Indexes for Assessing Coronary Stenosis Severity

**DOI:** 10.1155/2023/6461691

**Published:** 2023-06-29

**Authors:** Stephen Malmberg, Jörg Lauermann, Patric Karlström, Dario Gulin, Neshro Barmano

**Affiliations:** ^1^Department of Health, Medicine and Caring Sciences, Linköping University, Linköping, Sweden; ^2^Department of Internal Medicine, Ryhov County Hospital, Jönköping, Sweden

## Abstract

The adenosine-requiring physiological index fractional flow reserve (FFR) is the gold-standard method for determining the significance of intermediate lesions, while the resting full-cycle ratio (RFR) is a novel nonhyperaemic index without the need for adenosine administration. The aim of this study was to investigate the degree of concordance between RFR and FFR in indicating the need for revascularisation in patients with intermediate coronary lesions. This was a retrospective, registry-based study utilising data from the SWEDEHEART registry. Patients treated at Ryhov County Hospital in Jönköping, Sweden, between the 1^st^ of January 2020 and the 30^th^ of September 2021, were included. The degree of correlation and concordance between RFR and FFR was determined, both when used with a single cut-off (significant stenosis if RFR ≤0.89) and with a hybrid approach (significant stenosis if RFR ≤0.85, not significant if RFR ≥0.94, and FFR measurement when RFR was in the grey zone 0.86–0.93). The study population consisted of 143 patients with 200 lesions. The overall correlation between FFR and RFR was significant (*r* = 0.715, *R*^2^ = 0.511, *p* ≤ 0.01). A strong correlation was seen for lesions in the left anterior descending artery (LAD) and the left circumflex artery (LCX) (*r* = 0.748 and 0.742, respectively, both *p* ≤ 0.01), while the correlation in the right coronary artery (RCA) was moderate (*r* = 0.524, *p* ≤ 0.01). The overall concordance between FFR and RFR using a single cut-off was 79.0%. With a hybrid cut-off approach, the degree of concordance was 91%, with no need of adenosine in 50.5% of the lesions. In conclusion, there was a strong correlation and a high degree of concordance between FFR and RFR in determining the significance of a stenosis. The use of a hybrid approach could improve the identification of physiologically significant stenoses while minimising the use of adenosine.

## 1. Introduction

Coronary artery disease (CAD) is a leading cause of death worldwide [1], with a prevalence of approximately 3.8% of the Swedish population and an incidence of 158 cases per 100,000 people in Western Europe [2, 3]. The most common cause of CAD is atherosclerotic lesions, which encroach on the lumen of coronary vessels, restricting coronary blood flow and causing an imbalance in the demand and supply of oxygen to the myocardium. The degree to which an atherosclerotic lesion causes a reduction in the diameter of a coronary vessel is, however, not a wholly reliable predictor of the extent of the myocardial ischaemia that it causes.

An intermediate coronary stenosis is defined as a stenosis in which the luminal diameter is reduced by 40–70% [4]. While revascularisation in vessels with intermediate lesions has the potential to improve the patient's performance status, reduce mortality, and improve anginal symptoms, it can also lead to worse outcomes than if the lesion had been left untreated [5–7]. Correct identification of those intermediate stenotic lesions that cause myocardial ischaemia and therefore will benefit from revascularisation is thus essential to ensure the best possible clinical outcomes.

Estimating the severity of intermediate stenotic lesions through visual assessment by angiography has clear limitations, and even among experienced interventional cardiologists, there is a significant degree of intraobserver and interobserver variability in the classification of such lesions [8, 9]. As such, in patients with multivessel CAD, guiding the use of percutaneous coronary intervention (PCI) in intermediate lesions by angiography alone has been shown to be inferior, in terms of avoiding major adverse events, to assessment using physiological indexes such as fractional flow reserve (FFR) in combination with angiography [10].

FFR is an invasive catheter-based method for determining the physiological significance of a coronary stenosis and is the current gold-standard method for guiding the use of PCI in intermediate lesions in patients with multivessel CAD or without evidence of ischaemia in noninvasive testing [11]. FFR is defined as the ratio of distal mean coronary pressure to the mean aortic pressure (Pd/Pa) during hyperaemia, commonly induced by adenosine. Recent studies, however, have shown nonhyperaemic indexes such as the instantaneous wave-free ratio (iFR) to be highly viable alternative methods for assessing the need for revascularisation [12, 13], while at the same time, reducing discomfort for patients, cost, and time expenditure and avoiding the potential side effects that hyperaemia entails [14–16]. Some of the common side effects, though short-lasting, are chest pain and bronchospasm [17]. Some other uncommon side effects include ventricular asystole, bifascicular block, complete heart block, and sinus arrest [17–19].

The resting full-cycle ratio (RFR) is a new nonhyperaemic index for measuring the significance of lesions in coronary vessels. RFR is defined as the lowest ratio of the distal pressure to the aortic pressure (Pd/Pa) during the entire cardiac cycle and is measured in a resting condition without induced hyperaemia. RFR and iFR have previously been shown to correlate closely [20–22]. There is, however, still a need for further studies to validate RFR directly against FFR, as only a few studies on the concordance between RFR and FFR have been conducted to date [23, 24].

The standard cut-offs for FFR and RFR are 0.80 and 0.89, respectively. Below or at the cut-offs, revascularisation is indicated, and above the cut-offs, deferral of revascularisation in favour of medicinal treatment is indicated [11, 21]. In a retrospective study, RFR has been shown to have a diagnostic accuracy of 81.3% compared with FFR when using the single cut-off approach (RFR ≤0.89) [21]. As stated previously, iFR and RFR have been shown to correlate closely and are diagnostic equivalents [21]. For iFR, studies have been conducted in which iFR is used with a hybrid approach, and these indicate that such an approach may be employed to increase its diagnostic accuracy when compared to FFR. When a hybrid approach is used, where iFR values at or below 0.85 indicate revascularisation, values in the grey zone range of 0.86–0.93 indicate that FFR should be used, and values above 0.93 indicate deferral, the diagnostic accuracy rises to 94.2%, while at the same time precluding the need of adenosine in 69.1% of stenoses [25]. Considering that iFR and RFR have been shown to be diagnostic equivalents, it can be assumed that using a hybrid approach with RFR would yield similar results, and indeed, some preliminary, unpublished, results indicate this to be the case [26]. However, there do not seem to be any published studies that have compared the diagnostic accuracy of RFR in relation to FFR when RFR is interpreted using the hybrid approach.

The aim of this study was to investigate the degree of concordance between RFR and FFR in indicating the need for revascularisation in patients with intermediate coronary lesions.

## 2. Materials and Methods

### 2.1. Data Collection and Study Population

The Swedish Web-system for Enhancement and Development of Evidence-based care in Heart disease Evaluated According to Recommended Therapies (SWEDEHEART) registry is a Swedish national quality registry whose purpose is to improve the treatment and health outcomes for patients with acute coronary syndrome or who are undergoing angiography and angioplasty. SWEDEHEART contains several subregistries, including the Swedish Coronary Angiography and Angioplasty Registry (SCAAR). The data on patients contained within SCAAR include among other things patient demographics, baseline characteristics, which procedures were performed, the findings of assessment by angiography and FFR measurement, the placement of stents, and incurred complications [27].

**Figure 1:**
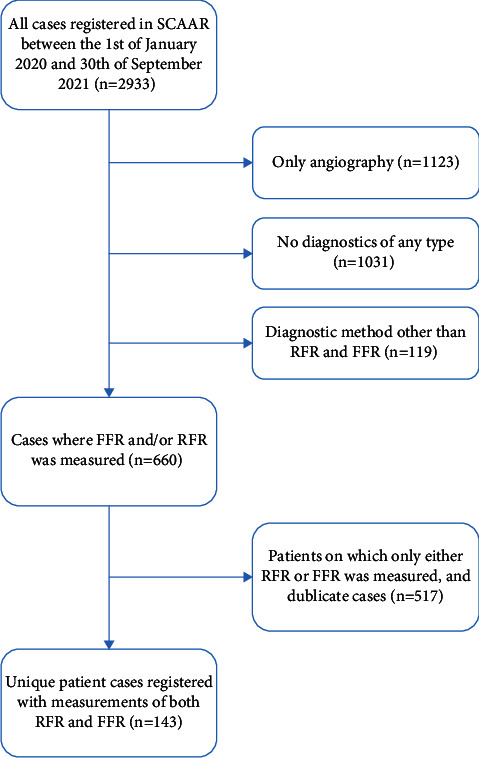
Flowchart describing the selection of the study population. FFR, fractional flow reserve; RFR, resting full-cycle ratio; SCAAR, Swedish Coronary Angiography and Angioplasty Registry.

The database used in this study included all patients that underwent coronary angiography at Ryhov County Hospital in Jönköping, Sweden, and that were registered in the SCAAR registry between the 1^st^ of January 2020 and the 30^th^ of September 2021. A total of 2933 events were registered. Patients, who only underwent angiography, did not undergo diagnostics of any type, were assessed with a diagnostic method other than FFR or RFR, or for whom only measurement of RFR or FFR was carried out, were excluded. With these exclusions, the final study population consisted of 143 patients (Figure 1). Assessments of stenotic lesions in more than one vessel were carried out for some patients. Some lesions were assessed pre- and post-PCI. Only lesions for which both RFR and FFR were measured were included, resulting in a total of 200 measurements of both FFR and RFR.

### 2.2. Measurement of the Physiological Indexes

Using either a radial or a femoral approach, a 6 French (F), or more seldom a 5F, guiding catheter was advanced to the coronary ostium appropriate for the vessel that was being assessed. The pressure wire and the guiding catheter were then calibrated with the atmospheric pressure as the zero point. The pressure wire was then advanced to a position just distal to the guiding catheter. Positioned like this, the pressure readings from the guiding catheter and pressure wire were manually equalised through the console. The pressure wire was then advanced past the stenosis. For RFR, the measurement was done with the patient in a resting state with the guiding catheter and pressure wire positioned as described. Before measurement of FFR can be done, hyperaemia must be induced, normally through intravenous or intracoronary administration of adenosine. At the catheterisation laboratory in Jönköping, intracoronary administration is more commonly used. A solution of 5–8 ml of 40 *μ*g/ml adenosine was administered in the left coronary artery and 3–5 ml in the right coronary artery (RCA), according to which the vessel was being assessed. In some cases, such as left main stenosis or osteal stenosis of the RCA, adenosine was instead administered intravenously at a rate of 140 *μ*g/kg/min. For both RFR and FFR, intracoronary nitroglycerine was administered. The distal pressure was measured by a 0.014-inch pressure guidewire (Abbott™ Pressurewire X, Abbot Vascular, Santa Clara, CA, USA), and the aortic pressure was measured by the guiding catheter. Measurements of both indexes were done using the Quantien™ system (Abbot Vascular, Santa Clara, CA, USA).

### 2.3. Statistics

Continuous variables were expressed as means ± SD if normally distributed and as medians with an interquartile range from the 25^th^ to the 75^th^ percentiles if not normally distributed. Categorical variables were expressed as numbers and percentages of cases. Differences between groups were tested for using the chi-squared tests for categorical variables. The degree of correlation between FFR and RFR was analysed using Pearson's correlation coefficient and the goodness of fit (coefficient of determination, *R*^2^) through simple linear regression analysis. Concordance, in this study, was defined as the number of correctly identified lesions divided by the total number of lesions. Receiver operating characteristic (ROC) curve analysis was performed to examine the agreement of RFR and RFR using FFR ≤0.80 as the reference standard. The sensitivity (Sn), specificity (Sp), positive predictive value (PPV), and negative predictive value (NPV) of RFR using FFR as the reference standard were calculated. A subgroup analysis was performed based on the vessel assessed. *p* values <0.05 were considered significant. All statistical analyses were performed using the SPSS 26 software. All analyses were performed using IBM SPSS, version 26 (IBM Inc., Armonk, NY, USA).

### 2.4. Ethical Approval

The study was performed in accordance with the Declaration of Helsinki and was approved by the Swedish Ethical Review Authority (Dnr. 2021-05980-01) [28].

## 3. Results

### 3.1. Patient Characteristics

Patient characteristics are presented in Table 1 and lesion characteristics are presented in Table 2.

### 3.2. Correlation and Diagnostic Precision of RFR Compared to FFR

**Figure 2:**
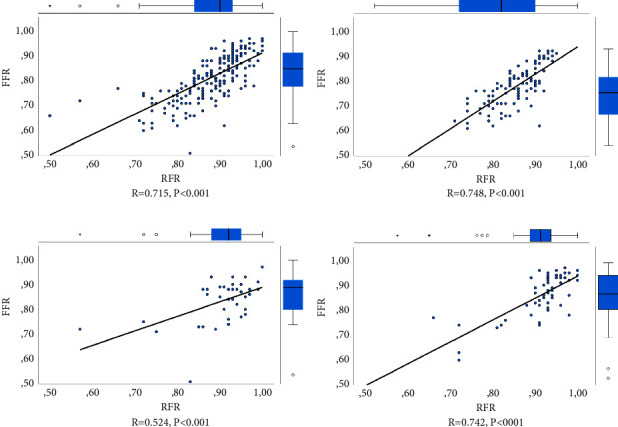
Correlation between FFR and RFR. (a–d) Scatterplots with linear regression lines for RFR plotted against FFR for all vessels, LAD, RCA, and LCX, respectively. FFR, fractional flow reserve; LAD, left anterior descending artery; LCX, left circumflex artery; RCA, right coronary artery; RFR, resting full-cycle ratio. (a) *R* = 0.715, *p* < 0.001, (b) *R* = 0.748, *p* < 0.001, (c) *R* = 0.524, *p* < 0.001, and (d) *R* = 0.742, *p* < 0001.

**Figure 3:**
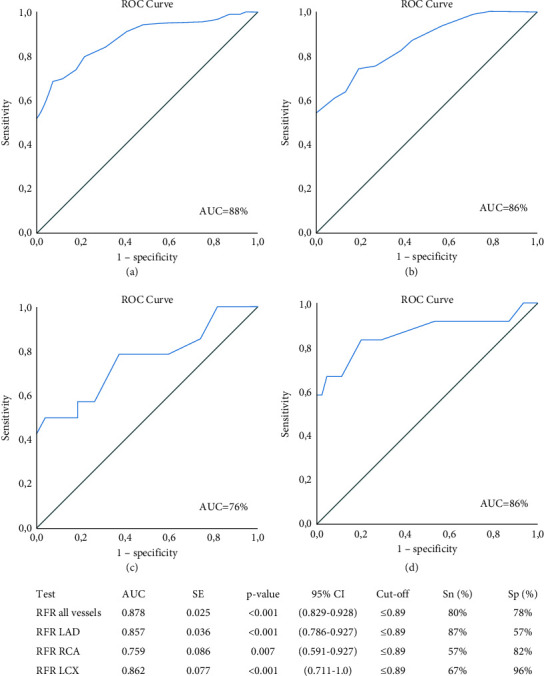
ROC curves with accompanying table showing the diagnostic accuracy of RFR for detecting FFR <0.80. (a–d) ROC curves for all vessels, LAD, RCA, and LCX, respectively. AUC, area under the curve; CI, confidence interval; FFR, fractional flow reserve; LAD, left anterior descending artery; LCX, left circumflex artery; RCA, right coronary artery; RFR, resting full-cycle ratio; ROC, receiver operating characteristic; SE, standard error; Sn, sensitivity; Sp, specificity.

Linear regression analysis showed a strong linear relationship between FFR and RFR when all measurements in all vessels were considered together (*r* = 0.715, *R*^2^ = 0.511, *p* ≤ 0.001; Figure 2). A similarly strong correlation was seen for lesions in LAD and LCX (*r* = 0.748 and 0.742, respectively, *p* ≤ 0.001 for both), while the correlation in the RCA was moderate (*r* = 0.524, *p* ≤ 0.001; Figure 2). ROC analysis showed an area under the curve (AUC) of 0.878 (95% CI 0.829–0.928, *p* ≤ 0.001), 0.857 (95%CI 0.786–0.927, *p* ≤ 0.001), 0.862 (95% CI 0.711–1.0, *p* ≤ 0.001), and 0.759 (95% CI 0.591–0.927, *p*=0.007), for all lesions and lesions in LAD, LCX, and RCA, respectively (Figure 3).

Overall concordance between FFR and RFR, using the single cut-off (RFR ≤0.89, FFR ≤0.80), was 79.0% for all lesions (Table 3). The lowest degree of concordance was found for RCA lesions and the highest degree of concordance was found for lesions in the LCX (Table 3). The Sn, Sp, PPV, and NPV for RFR using a single cut-off are shown in Table 4.

### 3.3. Hybrid Approach

Using a hybrid cut-off approach as described above, the degree of concordance with FFR was 91.0% (Table 5). Of the 200 lesions, 101 (50.5%) were outside the grey zone, not in need of assessment with adenosine. The number of lesions that would be wrongly classified according to FFR was nine (4.5%), five of those (2.5%) as significant and four (2.0%) as nonsignificant (Table 5). The Sn, Sp, PPV, and NPV were 93.1%, 88.4%, 91.5%, and 90.5%, respectively.

## 4. Discussion

The main findings of this retrospective registry-based study of patients undergoing physiological assessment of intermediate coronary artery stenoses are that there is a strong linear relationship between FFR and RFR and a high degree of agreement between the two methods in determining the significance of a stenosis. Using a hybrid approach, Sn and Sp increased by 13.3 and 10.0 percentage points, respectively, and in half of all the measurements, it was possible to omit adenosine.

### 4.1. Correlation and Diagnostic Precision of FFR vs RFR

There was a strong linear relationship between RFR and FFR for all vessels considered together (*r* = 0.715, *R*^2^ = 0.511, *p* ≤ 0.001) as well as for each vessel separately, except for the RCA, where only a moderately strong relationship was found. The results are very similar to previous studies, in which the corresponding values for overall correlation between RFR and FFR in all vessels were *r* = 0.746, *R*^2^ = 0.557, *p* < 0.001 and *r* = 0.770, *R*^2^ = 0.592, *p* < 0.001 [21, 23]. The ROC curve analysis, using FFR as the reference standard, showed that RFR had good diagnostic precision when considering all the measurements together, as well as for each coronary vessel separately. The AUC in all vessels was 0.878 (95% CI 0.828–0.933), which also is in line with the previous studies in which the AUC values were 0.881 (95% CI 0.856–0.906, *p* < 0.001) and 0.870 (96% CI 0.824–0.916, *p* < 0.001), respectively [21, 23]. A test with an AUC value of 0.8-0.9 is generally considered as having excellent discriminatory ability, while tests with values between 0.7 and 0.8 are considered acceptable [29].

### 4.2. Concordance of FFR and RFR

The concordance between RFR and FFR in this study was 79.0% for all lesions, a result very similar to those of previous studies where the concordance was 81.4%, 80.5%, and 81.3% [21, 23, 24]. The way that diagnostic accuracy varied among the vessels differed in this study compared to previous studies. In the present study, the diagnostic accuracy values for LAD, RCA, and LCX were 75.5%, 73.2%, and 89.5%, respectively. In a previous study, the corresponding numbers were 73.7%, 86.9%, and 88.2% [23]. While the degree of concordance was similar in LAD and LCX, the diagnostic accuracy for RCA was lower by a fair margin in the present study. Though the reason for this discrepancy is unclear, a potential explanation could be differences in the study populations and a lower number of studied lesions in RCA in the present study compared to the number of studied lesions in LAD and LCX. Perhaps if the number of assessed lesions in the RCA had been greater, the size of the difference in diagnostic accuracy in RCA would be more akin to the much smaller difference seen between the studies for diagnostic accuracy in LAD and LCX. Overall, the finding of high overall concordance between RFR and FFR in this study and in previous studies suggests that RFR is a physiological index with a high utility for identifying significant lesions in accordance with FFR [20, 21, 23, 24].

### 4.3. Discordance between RFR and FFR

The overall rate of discordance between RFR and FFR was 21.0%, with the values for LAD, RCA, and LCX being 24.5%, 26.8%, and 10.5%, respectively. In the cases where FFR and RFR disagree, evidence from a study by Lee et al. shows that deferral, both in high FFR/low RFR and low FFR/high RFR groups, is safe and not associated with an increased risk of vessel-oriented composite outcomes (VOCO) after two years when compared to the high FFR/high RFR group. VOCO, as defined in the study, included outcomes such as cardiac death, myocardial infarction related to the assessed vessel, and revascularisation related to the assessed vessel [22]. The findings suggest certain complementariness between FFR and resting such as RFR. As resting and hyperaemic indexes are measured under different conditions, they, in part, give insights into different aspects of the flow conditions in the lesion. More complete information on the significance of the lesion could thus be gleaned from using FFR and resting indexes such as RFR and iFR in conjunction [30].

### 4.4. Hybrid Approach

The current European Society of Cardiology guidelines rate the use of FFR and iFR measurements to guide revascularisation with an evidence level of A and give both a Class 1A recommendation [11]. The use of other physiological indexes such as RFR has yet to be classified. Since RFR has been shown to be diagnostically equivalent to iFR, with a concordance of 97.4% and a high correlation (*R*^2^ = 0.985) [21] and iFR has been shown to be noninferior to FFR in terms of major cardiac events [12], it can be expected that an RFR-guided revascularisation strategy would yield similar results in comparison to a FFR-guided revascularisation strategy. However, since to date there has been no randomised controlled study demonstrating the noninferiority of RFR compared to FFR in terms of clinical outcome, FFR is regarded as the gold standard.

An alternative use of RFR may thus be to use it as a complement to FFR, with a hybrid approach. With a hybrid approach, the concordance in this study between RFR and FFR was 91.0%, with values for Sn, Sp, PPV, and NPV being 93.1%, 88.4%, 91.5%, and 90.5%, respectively. This indicates that using a hybrid approach for RFR would result in a high degree of concordance with FFR (91.0%), while at the same time reducing discomfort for patients, reducing cost, and saving time as 50% of patients would be spared having to be administered adenosine, with the potential discomfort this entails [13, 17–19].

Had the hybrid approach been used, nine lesions (4.5% of all measurements) outside the grey zone would have been wrongly categorised according to FFR. This margin of error could reasonably be considered too large in a clinical situation in which an accurate assessment of the lesion is of high importance. However, it is worth noting that FFR is not the only measure of truth in determining the significance of a stenosis and that it may well be that RFR, as stated earlier, is an equally good physiological index for independently determining the significance of a stenosis, in terms of clinical outcome. In one study, the diagnostic performance of FFR and iFR in terms of identifying myocardial ischaemia was compared [31]. The extent of myocardial ischaemia was determined through assessment with coronary flow reserve and assessment of the hyperaemic myocardial blood flow by use of N-ammonia positron emission tomography. This study found that iFR had better agreement than FFR with the measures of myocardial ischaemia. Given the diagnostic equivalence between iFR and RFR, one could thus argue that RFR is better at identifying myocardial ischaemia than FFR [31].

### 4.5. Strengths and Limitations of the Study

The main strengths of this study are that it is based on real life data and that it directly compares RFR with FFR and thus contributes to the understanding of a topic on which relatively few studies have been conducted.

Some of the limitations of this study are the population size, especially concerning the number of measurements of the physiological indexes in RCA and LCX, and that this is a single centre study. The design of the study, being retrospective and registry-based, also means that the way in which the physiological indexes were measured was not as systematic as it would have been in the context of a randomised controlled clinical trial. Therefore, there may have been slight variations in the way the different interventional cardiologists carried out the measurements, as there was not a strict study protocol for them to adhere to. Furthermore, it is custom in our centre to use physiological assessment in nonculprit vessels only, in the setting of acute coronary syndrome, but due to the design of the study, data on whether measurements were made in culprit vessels as well are lacking. The smaller size of the study also precludes certain subgroup analysis from being done in any meaningful way. Another limitation of this study is that the dataset did not include information on whether RFR was measured in systole or diastole. As one of the theoretical selling points of RFR compared to iFR is its nondiscriminatory way of analysing the whole cardiac cycle to find the point at which the flow is the most impaired [21], subgroup analysis based on the location of RFR in the heart cycle may have yielded valuable information.

## 5. Conclusion

In this retrospective registry-based study, there was a strong correlation, and a high degree of agreement in determining the significance of a stenosis, between RFR and FFR. The use of a hybrid approach further improved the identification of physiologically significant stenoses while minimising the use of adenosine. Future randomised controlled studies are needed to find out whether RFR, like iFR, is noninferior to FFR in terms of preventing major adverse cardiac events.

## Figures and Tables

**Table 1:** Patient characteristics (*n* = 143).

Variables	Values
Male sex	114 (79.7%)
Age (y)	69 ± 10
BMI (kg/m^2^)	27.9 ± 4.5
S-creatinine (mmol/L)	79.5 ± 18.0
Hypertension	107 (74.8%)
Diabetes	43 (30.0%)
Lipid lowering agent	110 (76.9%)
History of smoking or current smoker	87 (60.8%)
Previous MI	69 (48.3%)
Previous PCI	72 (50.3%)
Previous CABG	7 (4.9%)
Previous stroke	6 (4.2%)
Peripheral arterial disease	3 (2.1%)

*Indication for coronary angiography*	
NSTEMI	31 (21.7%)
Unstable angina pectoris	14 (9.8%)
Stable angina pectoris	28 (19.6%)
Staged procedure, completion of earlier PCI	34 (23.8%)
Arrhythmia	7 (4.9%)
Heart failure, cardiomyopathy	8 (5.6%)
Valvular heart disease	18 (12.6%)
Unclear chest pain	1 (0.6%)
Silent ischaemia	1 (0.6%)
Others	1 (0.6%)

*Note.* Values are expressed as mean ± SD or as *n* (%). BMI, body mass index; CABG, coronary artery bypass graft surgery; MI, myocardial infarction; NSTEMI, non-ST-elevation myocardial infarction; PCI, percutaneous coronary intervention; *n*, number; *y*, years.

**Table 2:** Lesion characteristics (*n* = 200 lesions).

*Vessels*	
LAD	98 (49.0%)
RCA	41 (20.5%)
LCX	57 (28.5%)
LM	4 (2.0%)

*Physiological measurements*	
FFR	0.81 ± 0.09
RFR	0.88 ± 0.08
FFR ≤0.80	89 (44.5%)
RFR ≤0.89	95 (47.5%)

*Note.* Values are expressed as mean ± SD or as *n* (%). FFR, fractional flow reserve; LAD, left anterior descending artery; LCX, left circumflex artery; LM, left main; RCA, right coronary artery; RFR, resting full-cycle ratio.

**Table 3:** Concordance between RFR and FFR.

	High FFR/high RFR	High FFR/low RFR	Low FFR/high RFR	Low FFR/low RFR	Concordance (*n* (%))	Discordance (*n* (%))
All vessels	87 (43.5%)	24 (12.0%)	18 (9.0%)	71 (35.5%)	158 (79.0%)	42 (21.0%)
LAD	21 (21.4%)	16 (16.3%)	8 (8.2%)	53 (54.1%)	74 (75.5%)	24 (24.5%)
RCA	22 (53.7%)	5 (12.2%)	6 (14.6%)	8 (19.5%)	30 (73.2%)	11 (26.8%)
LCX	43 (75.4%)	2 (3.5%)	4 (7.0%)	8 (14.0%)	51 (89.5%)	6 (10.5%)

*Note.* Values are expressed as *n* (%). FFR , values ≤0.80 indicate low FFR and >0.80 high FFR. RFR, values ≤0.89 indicate low RFR and >0.89 high RFR. Concordance was defined as the number of correctly identified lesions divided by the total number of lesions, while discordance was defined as the number of incorrectly identified lesions divided by the total number of lesions. FFR, fractional flow reserve; LAD, left anterior descending coronary artery; LCX, left circumflex coronary artery; RCA, right coronary artery; RFR, resting full-cycle ratio.

**Table 4:** Sensitivity, specificity, positive predictive value, and negative predictive value of RFR.

	Sensitivity (%)	Specificity (%)	Positive predictive value (%)	Negative predictive value (%)
All lesions	79.8	78.4	74.7	82.9
LAD	86.9	56.8	76.8	72.4
RCA	57.1	81.5	61.5	78.6
LCX	66.7	95.6	80.0	91.5

*Note.* Values are expressed as *n* (%). LAD, left anterior descending coronary artery; LCX, left circumflex coronary artery; RCA, right coronary artery.

**Table 5:** Classification of lesions according to the hybrid approach.

	RFR treat ≤0.85	RFR 0.86–0.93 “grey zone”	RFR defer ≥0.94
FFR defer	5 (2.5%)	68 (34.0%)	38 (19.0%)
FFR treat	54 (27.0%)	31 (15.5%)	4 (2.0%)

*Note.* Values are expressed as *n* (%). FFR, fractional flow reserve; RFR, resting full-cycle ratio.

## Data Availability

The data used in this study originates from the SWEDEHEART registry and contains sensitive patient information. The dataset analysed in this study is not publicly available due to Swedish patient privacy and secrecy laws and due to ethical restrictions regarding the current analysis (the Swedish Ethical Review Authority, Dnr. 2021-05980-01). Requests for access to unidentified data can be made to the corresponding author.
